# Tailoring Thermoplastic Whey Protein with Polyethylene Glycol and Calcium Stearate: A Systematic Design of Experiments Investigation of Structure, Thermal Behavior, and Rheology

**DOI:** 10.1021/acsomega.6c06950

**Published:** 2026-10-03

**Authors:** Amanda Maria Blumrich, Pedro Arthur Lopes Saad Meirelles, Antony Barbosa, Marina Fernandes Cosate de Andrade

**Affiliations:** Departament of Materials Engineering and Bioprocess, School of Chemical Engineering, 28132 Universidade Estadual de Campinas Avenue Albert Einstein, 500 - Cidade Universitária Zeferino Vaz, Barão Geraldo, Campinas, 13083-872 São Paulo, BR

## Abstract

The growing environmental concern related to plastic materials has driven the search for sustainable alternatives. In this context, whey protein isolate (WPI) has emerged as a promising option due to its adequate oxygen barrier properties for food packaging. However, WPI lacks mechanical resistance, requiring plasticization to increase protein chain mobility, flexibility, and processability. WPI plasticized during its denaturation is called thermoplastic whey protein isolate (WPIT). This study evaluates the WPIT modification using a design of experiments (DoE) with two factors and a center point: calcium stearate, a largely commercially used lubricant, and the plasticizer poly­(ethylene glycol) (PEG). To assess the occurrence of plasticization and the proposed stabilization, the structural, rheological, and thermal characteristics of the modified WPIT were investigated. Fourier-transform infrared spectroscopy (FTIR) showed no significant increase in the α helix secondary structure, suggesting incomplete plasticization. The parallel plate rheology revealed that the complex viscosity followed power law behavior, and there was no Newtonian plateau. WPIT samples with calcium stearate and without PEG, and the center point presented crossover of storage and loss moduli in the terminal zone. The sample with maximum calcium stearate and no PEG showed evidence of cross-linking. Differential scanning calorimetry (DSC) analysis did not allow for the identification of the glass transition temperature (*T*_g_) for WPIT samples. Thermogravimetric analysis (TGA) results indicated decreased thermal stability in the presence of PEG, likely due to its plasticizing effect. The results suggested that the presence of calcium ions may have promoted possible cross-linking between the protein chains.

## Graphical abstract

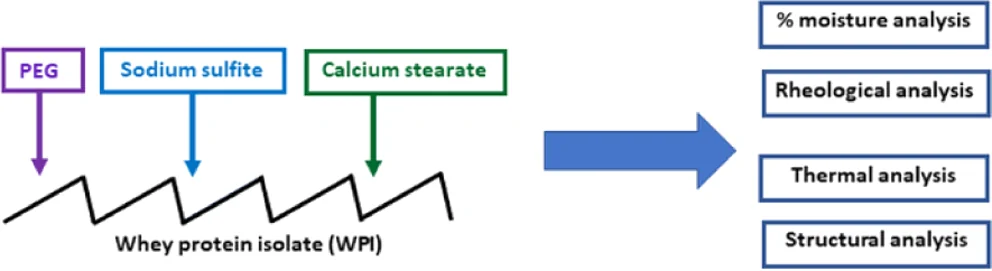

## 1 Introduction

Fossil carbon sources constitute the majority of the raw materials processed to form plastics for various applications.^1^ Global plastic waste generation went from 176 million tons in 2000 to 353 million tons in 2019, with only 9% being recycled, 19% incinerated, 50% landfilled, and 22% classified as mismanaged, ending up in the environment.^2^ In this regard, the packaging industry, with particular emphasis on food packaging, has been a major source of debris, as its assets constitute mostly short-term usage items, but with materials of an extremely long-life span. Hence, plastics manufactured for this purpose, which are virtually of petrochemical origin, are continuously produced and shortly after discarded.^1^

A proposed solution has been to implement a complete or partial transition to renewable materials, as they do not offer the same environmental impact due to how fast they can be replenished after their immediate processing.^3^ From the array of biopolymers available to provide functionality as films designed for packaging, proteins constitute a prominent source. Plant-based and animal-based proteins are the two classes of these polymers that can be characterized by their unique features. The former, which includes sources such as wheat, soy, and pea, is considered cheaper and more accessible, having lower flexibility and tensile strength. As for the latter, gelatin, albumin, and whey protein are among the most studied candidates for this type of application. These proteins have oxygen barrier properties comparable to commercial polymers such as ethylene vinyl alcohol (EVOH), but do not offer the same water vapor barrier properties.^4^

Whey protein is alongside casein, one of the protein fractions present in milk, accounting for 20% of its total mass. Its two main constituents are β-lactoglobulin and α-lactoalbumin, globular proteins, with the first being the most abundant.^5^

Whey protein isolate (WPI) has adequate gas and water vapor barrier properties for food packaging, but for processing, it must be converted into thermoplastic whey protein isolate (WPIT) through thermal denaturation and the addition of plasticizers, such as poly­(ethylene glycol) (PEG), which increase the mobility of protein chains.^6,7^ Hence, stabilizing the electrostatic interactions between the chains, which prevent the protein from unfolding completely, is essential to ensure the flexibility of the material.^8^ The addition of vitamin E and PEG caused a plasticizing effect in WPIT, enhancing the contribution of α-helices in its secondary structure, and reducing the glass transition temperature of the protein phase. The thermal stability of WPIT was also improved by the presence of vitamin E.^9^

Another example of additives is metallic stearates, such as calcium stearate, which are considered metallic soaps and derived from long-chain fatty acids and metallic oxides.^10^ Their chemical structures consist of chains with a polar part (metallic carboxylate) and a nonpolar part (hydrocarbon).^11^ Due to their chemical structure, especially alkaline earth metal soaps, metallic stearates are commonly used as lubricating agents for various thermoplastic polymers.^12^ Calcium stearate, a commercially used additive in polymers, is proposed in this study to modify the WPIT in the presence of PEG. A design of experiments (DoE) factorial design 2^2^ with a center point evaluated the effect of the factors calcium stearate and PEG content on the structural, rheological, and thermal responses of the material. The combined effect of the two additives evaluated by DoE represents the novelty of this work, including the rheological tests to understand more about its influence on WPIT rheology.

## 2 Materials and Methods

### 2.1 Materials

For sample preparation, whey protein isolate (Hilmar 9010 Instantized Whey Protein Isolate - Hilmar Ingredients), poly­(ethylene glycol) 400 (Dinâmica Química Contemporânea Ltd.a.), anhydrous sodium sulfite (Dinâmica Química Contemporânea Ltd.a.), and calcium stearate (CEASIT SW–Bærlocher do Brasil S.A) were used.

### 2.2 Methods

The study was conducted according to a design of experiments (DoE) with two levels and two factors with a center point, totaling five samples, using Minitab Statistical Software version 17. Calcium stearate and PEG content were the factors. Their composition is sorted in Table 1.

**Table 1:** Composition of Each Formulation (in g)

sample name	PEG content	calcium stearate content
**4P0.03**	4	0.03
**0P0.01**	0	0.01
**8P0.05**	8	0.05
**8P0.01**	8	0.01
**0P0.05**	0	0.05

The WPI and sodium sulfite remained at 10 and 0.2 g, respectively, across all samples. Initially, the solids of the mixture were stirred together manually, followed by the liquids. The liquid combination was poured into the solids, and then they were all stirred manually to prevent any mass sticking to the walls of the recipient. Then, the sample was stirred by a mechanical agitator (model TE-039/1) by Tecnal, Brazil, with a high shear impeller at 540 rpm for 10 min.

The recipient was then immediately put under heating, alongside stirring with the same rotation. When the suspension reached 75 °C, calcium stearate was added. The mixture remained in a range of 75–85 °C in the next 45 min. After that, it was transferred to a Petri dish and stayed for 8 h in a vacuum oven (model TE-395) by Tecnal, Brazil, during 8 h at 300 mmHg and 40 °C.

After its removal, the sample was stored in a refrigerator for 7 days.

The five samples were milled in SPEX’s Sample Prep Freezer/Mill 6870. They were all subjected to 5 cycles of 2 min grinding at a temperature of −180 °C by adding liquid nitrogen. The next step was to freeze-dry the samples, aiming to remove the remaining moisture. A day before, they were stored in an ultrafreezer for 24 h at −88 °C. They were then immediately brought to the freeze-drier Liotop 10 of Liobras and stayed there for 24 h.

#### 2.2.1 Caracterization

To evaluate modifications in the secondary structure of the protein, the amide I region of the proteins, obtained by Fourier-transform infrared spectroscopy (FTIR), was deconvoluted according to Ramos et al. (2012). FTIR analysis was performed in triplicate using Thermo Scientific Nicolet iS50 FT-IR equipment. The spectra were obtained in attenuated total reflection (ATR) mode (germanium crystal), in the range of 4000–675 cm^–1^, with a resolution of 4 cm^–1^ and 128 scans per spectrum. This study was performed using the FitPeaks function of OriginPro 8.1 software, covering wavelengths from 1591 cm^–1^ to 1751 cm^–1^. The objective was to assess the changes in the secondary structures of the protein with the addition of PEG and calcium stearate. This analysis was performed in triplicate.

Storage and loss moduli, as well as complex viscosity, were obtained by a parallel plate rheometer, Discovery HR2 of Waters TA Instruments, at 160 °C in a range of 0.01 to 1000 rad/s during the frequency sweep. After the amplitude sweep, the device set a shear deformation of 0.05% with an angular velocity of 6.2 rad/s.

To investigate the thermal stability of the samples, thermogravimetric analysis (TGA) was performed by heating the samples from room temperature to 600 °C in an inert atmosphere at a rate of 10 °C/min, according to ASTM E2550–11.^13^ The analysis was conducted using TGA550 equipment from TA Analytics Waters.

To analyze the thermal transitions of the samples, the differential scanning calorimetry (DSC) was performed using the Mettler Toledo Microanalytical Balance model XPR2. Samples were cooled from 25 °C to −80 °C and then heated from −80 to 200 °C, with both processes conducted at a rate of 10 °C/min and in an inert atmosphere.

The moisture content of the samples was determined using a MOC63U Moisture Determinator (Shimadzu) at 105 °C.

## 3 Results and Discussion

For the structural analysis, the FTIR spectra is shown in Figures 1 and 2, which show absorption bands characteristic of each substance. Calcium stearate has absorption bands at 1542 cm^–1^ and 1576 cm^–1^, associated with the vibrations of COO^–^ groups coordinated with Ca^2+^.^10^ The amide II region (1400–1550 cm^–1^), with an absorption band at 1530 cm^–1^, appears in WPI and in all samples,^14^ making it difficult to distinguish between the protein and stearate absorption bands due to overlap. Amide I, with an absorption band at 1642 cm^–1^ (1600–1700 cm^–1^), is also present, indicating C=O and C–N vibrations.^14^ PEG is identified by an absorption band at 1100 cm^–1^ related to O–C–O esters,^15^ present only in samples with PEG (4P0.03, 8P0.05, and 8P0.01) and in pure PEG. Absorption bands at 3285 cm^–1^, attributed to O–H and N–H stretching, suggest the presence of water.^16^ In addition, the absorption band at 2868 cm^–1^ indicates stretching of the −CH_2_ groups of PEG, observed in the same samples containing this component.^17^

**Figure 1:**
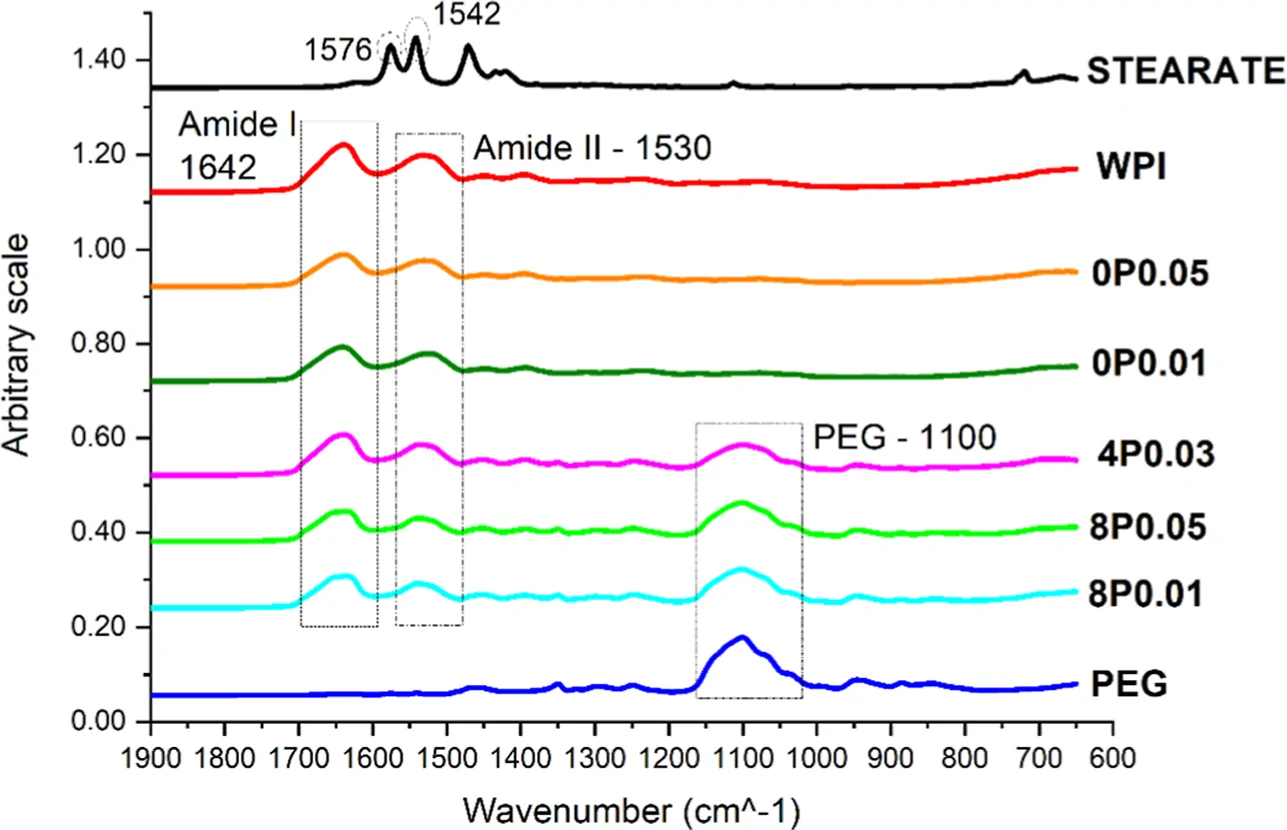
FTIR spectrum (1900–600 cm^–1^) obtained for the samples and pure components.

**Figure 2:**
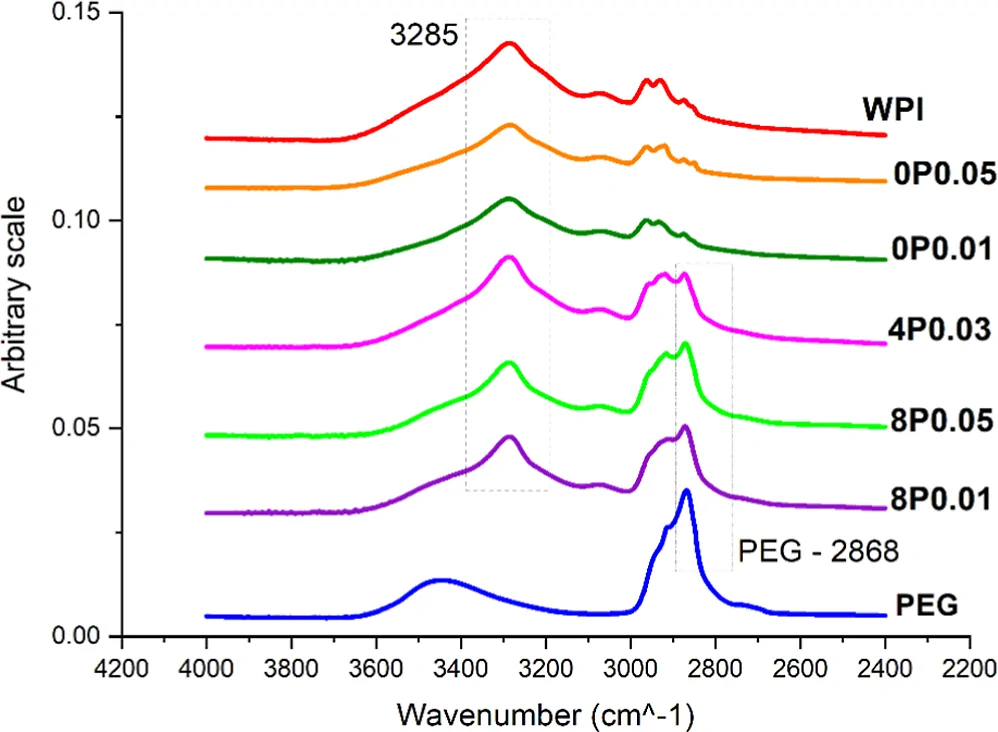
FTIR spectrum (4200–2200 cm^–1^) obtained for the samples and pure components.

The modifications in the secondary structure of the protein can be evaluated by the deconvolution of the amide I region. Absorption bands at 1623 cm^–1^ and 1633 cm^–1^ indicate extended beta sheets; at 1616 cm^–1^ and 1682 cm^–1^, antiparallel beta sheets; at 1644 cm^–1^ and 1652 cm^–1^, alpha helices; and at 1667 cm^–1^ and 1675 cm^–1^, turns.^14^ Table 2 shows the average contribution of each secondary structure in the amide I region for each sample.

**Table 2:** Contribution of Each Secondary Structure in the Amide I Region to the Samples

sample	PEG content (g)	Stearate content (g)	extended beta-sheets (%)	antiparallel beta-sheets (%)	α helix (%)	turns (%)
**WPI**	–	–	20%	34%	31%	15%
**4P0.03**	4.00	0.03	17%	35%	34%	15%
**0P0.01**	0.00	0.01	12%	38%	37%	13%
**8P0.05**	8.00	0.05	19%	30%	35%	16%
**8P0.01**	8.00	0.01	27%	31%	20%	22%
**0P0.05**	0.00	0.05	14%	37%	34%	15%

The main effects plots of PEG and calcium stearate for beta-sheet, α helix, antiparallel beta-sheet, and turns are presented in Figure 3 and reveal the influence of PEG and stearate content on each secondary structure in the analyzed samples. These results show that PEG has a greater influence on the extended beta-sheet (Figure 3a), antiparallel beta-sheet (Figure 3b), and turns (Figure 3c) structures when compared to the effect of calcium stearate content. For the α helix configuration (Figure 3d), the content of both PEG and calcium stearate shows a similar magnitude of influence, although the additives present opposite behavior. The addition of PEG in the presence of calcium stearate decreased the α helix content, which was not expected, as this conformation is favored during plasticization.^18^ The combination of PEG and vitamin E increased the α helix contribution for WPIT, indicating a plasticized effect.^9^

**Figure 3:**
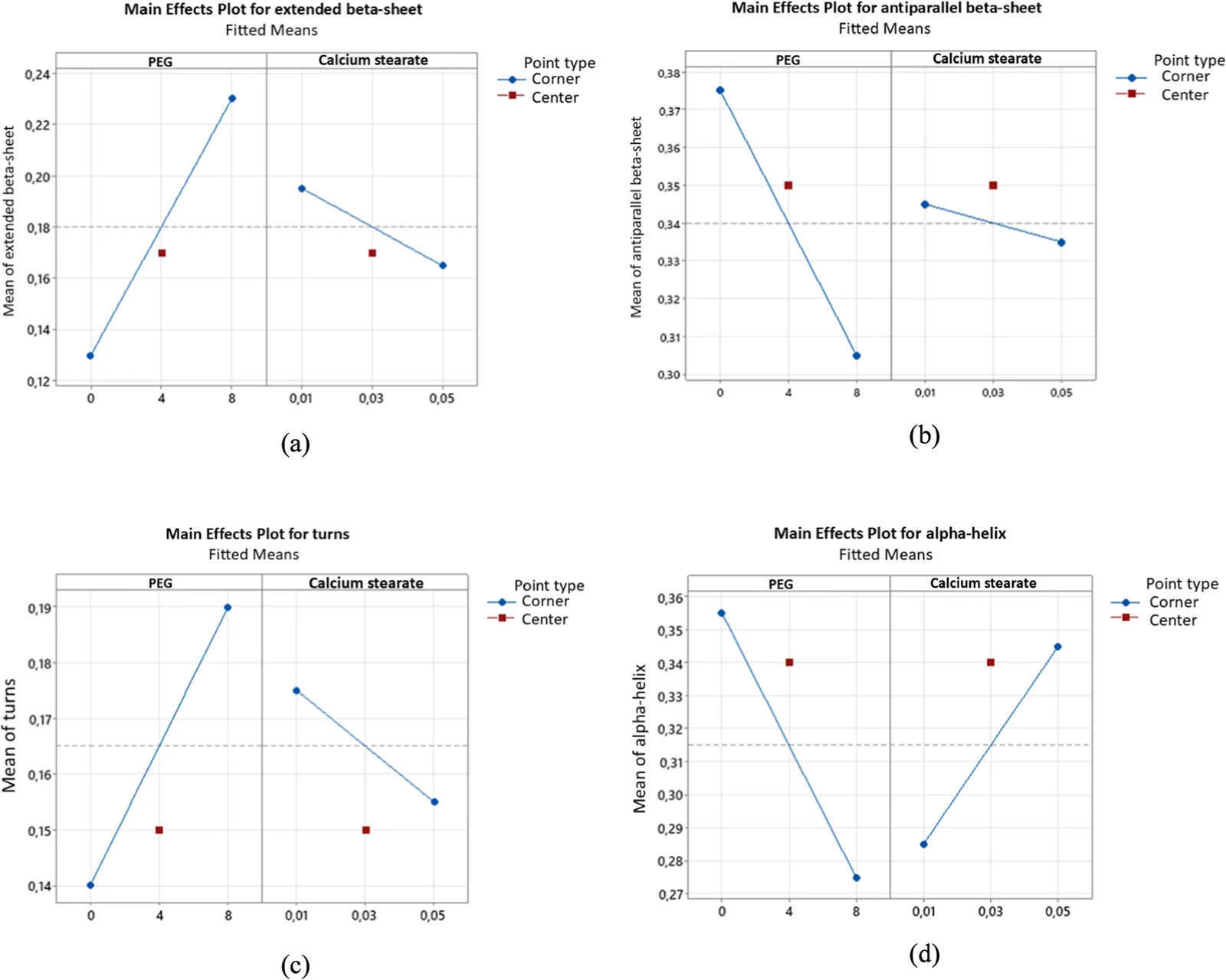
Main effects plots of PEG and calcium stearate factors for protein secondary structures: (a) extended beta-sheet; (b) antiparallel beta-sheet; (c) turns; (d) α helix.

The rheological curves for each sample are presented in Figure 4. In all samples, solid-like behavior predominates due to the superiority of the storage modulus (*G*′) over the loss modulus (*G*″),^19^ with its difference, hereafter referred to as GAP (GAP = *G*′–*G*″), increasing considerably with frequency. The samples exhibit a general tendency for the GAP to grow with an increase in angular frequency. At a molecular level, in very short time intervals or high frequencies, molecules do not have enough time to respond to shear stresses in the form of viscous dissipation and reorient themselves into lower energy conformations, which means times shorter than relaxation times.^20^ In this way, much of the energy is stored as elastic deformation.

**Figure 4:**
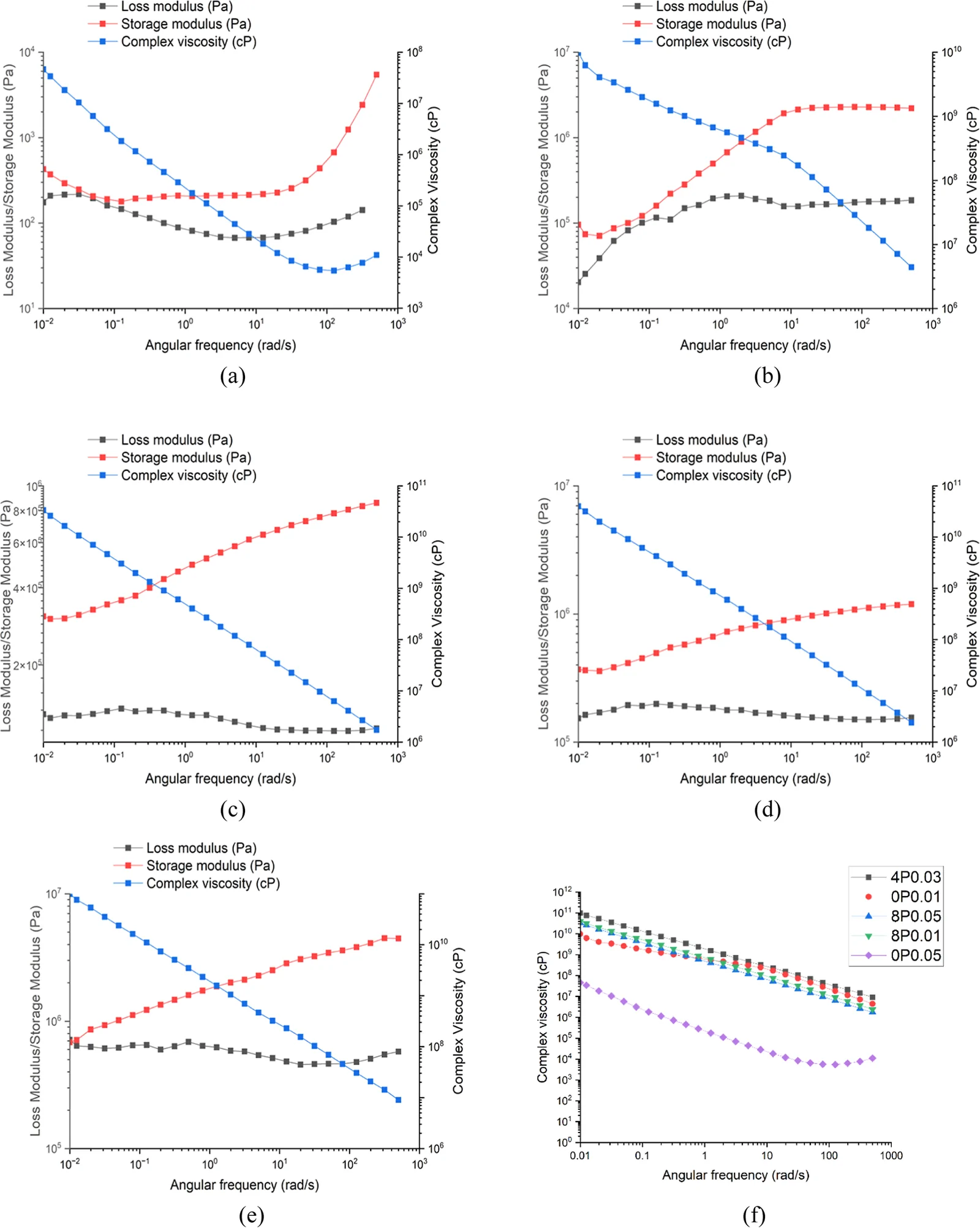
: Rheological curves of samples at 160 °C: (a) 0P0.05; (b) 0P0.01; (c) 8P0.05; (d) 8P0.01; (e) 4P0.03; (f) all samples.

For commercial thermoplastic polymers, there is a predominance of the loss modulus in relation to the storage modulus in the frequency range studied, if the material is above its melting temperature, except in the higher frequency regions, as shown for poly­(butylene adipate*-co-*terephthalate) (PBAT). At high frequencies, the WPIT showed a predominance of *G*″ at a temperature of 160 °C in the presence of PEG.^7^ The protein formulations in the presence of calcium stearate at 160 °C, even with the addition of PEG, did not show a predominance of the viscous component in the angular frequency sweep studied in this project. This indicates that the evaluated formulations did not produce a thermoplastic material, as corroborated by the FTIR results.

Table 3 presents the values of complex viscosity, storage modulus (*G*′), and loss modulus (*G*″) for samples at an angular frequency of 8.4 rad/s, which was used to process blends of PBAT/WPIT.^7^ Furthermore, there is a narrowing of the curves at low frequencies, with the occurrence closest to the desired inversion between *G*′ and *G*″. However, there was no predominance of *G*″ in any of the five cases examined, as shown by Figure 4.

**Table 3:** Parallel Plate Rheometry Results for an Angular Frequency of 8.4 rad/S

sample	*G*′(Pa)	*G*″(Pa)	complex viscosity (cP)	|*G*′–G″| (Pa)
4P0.03	2.5 × 10^6^	5.1 × 10^5^	3.1 × 10^8^	2.0 × 10^6^
0P0.05	2.1 × 10^2^	6.8 × 10^1^	2.7 × 10^4^	1.5 × 10^2^
0P0.01	2.0 × 10^6^	1.6 × 10^5^	2.4 × 10^8^	1.8 × 10^6^
8P0.05	6.2 × 10^5^	1.2 × 10^5^	7.7 × 10^7^	5.0 × 10^5^
8P0.01	9.0 × 10^5^	1.6 × 10^5^	1.1 × 10^8^	7.4 × 10^5^

The plasticization of WPI is expected to enhance chain mobility, leading to a decrease in viscosity.^6^ Nonetheless, as illustrated in Figure 4a, the 0P0.05 sample exhibited an increase in storage, loss, and complex viscosity moduli at higher frequencies, which may suggest the occurrence of protein cross-linking. Cross-linking refers to the formation of bonds between polymer chains, which restricts their rotational freedom and decreases the free volume by establishing a stable three-dimensional network. Several forms of cross-linking exist such as chemical, thermal and ultraviolet-induced, among others, each capable of producing different effects when applied under controlled conditions.^21^ The presence of cross-linking in a polymer structure increases its viscosity under high shear stress conditions.^22^

A power-law behavior without a Newtonian plateau in the terminal zone for complex viscosity was observed for samples containing PEG and could indicate the formation of gel.^23^ In the absence of a plasticizer, the reduction in viscosity is nonlinear. 0P0.05 presented an increase in complex viscosity at higher concentrations, probably due to cross-link formation.

Calcium stearate exhibits the greatest effect on GAP and complex viscosity compared to PEG at an angular frequency of 8.4 rad/s, as shown in the main effects plots in Figure 5a and c. There is no interaction between calcium stearate and PEG at an angular frequency of 8.4 rad/s, as shown in Figure 5b and d. Stearate can decrease the viscosity of polymers in the molten state, including polymers with polar groups in their chain, such as poly­(3-hydroxybutyrate).^24^ The most pronounced decrease in complex viscosity was 0P0.05, as is shown in Figure 4f. Calcium stearate’s influence on complex viscosity was predominant, as shown in Figure 5c, and it may have acted as a lubricant in the absence of a plasticizer, probably due to the stearate part of the molecule.

**Figure 5:**
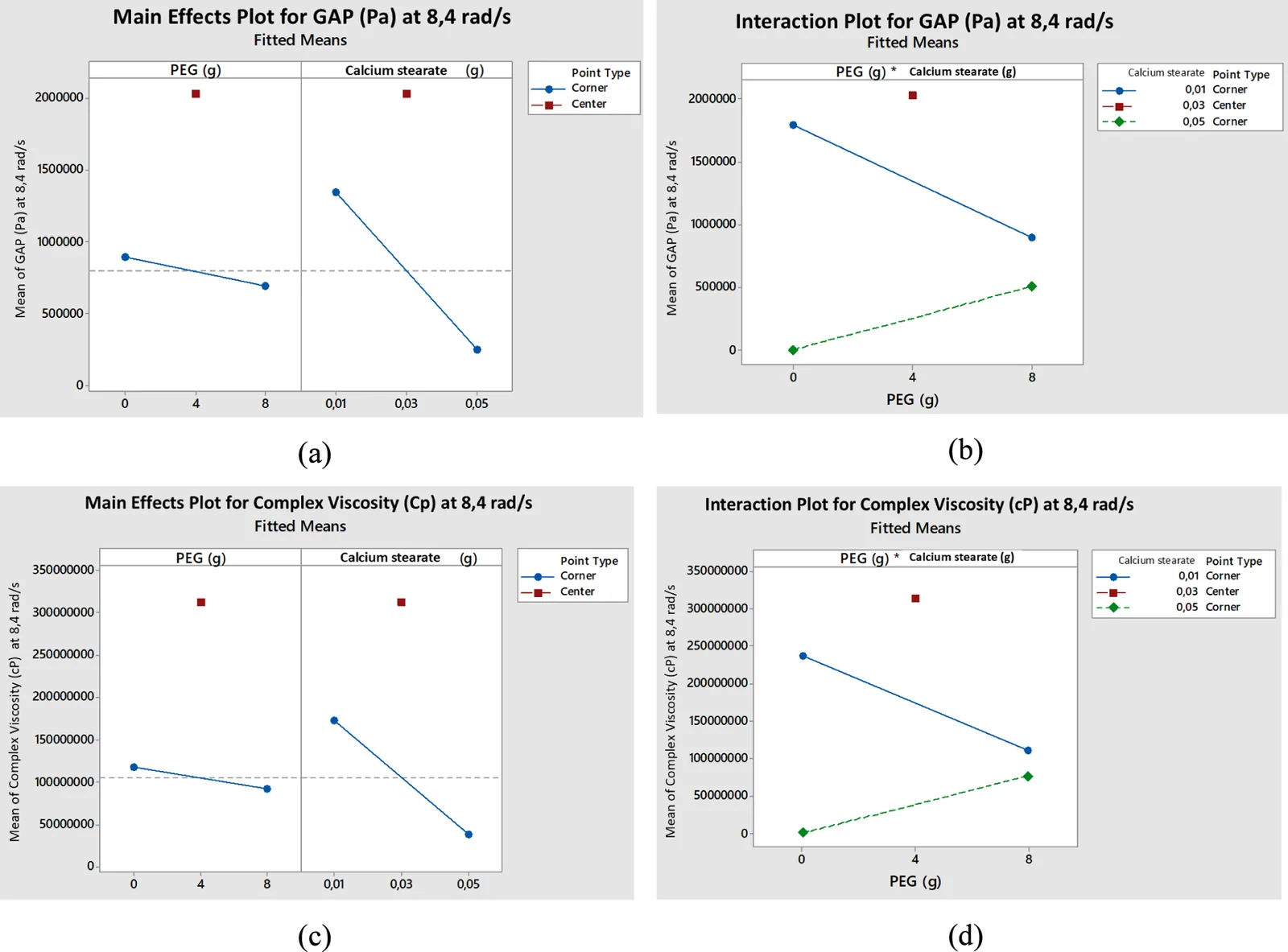
(A) Main effects plot of calcium stearate and PEG factors for GAP (in Pa); (b) Interaction plot of calcium stearate and PEG factors for GAP (in Pa); (c) Main effects plot of calcium stearate and PEG factors for complex viscosity (in cP); (d) Interaction plot of calcium stearate and PEG factors for complex viscosity (in cP).

However, the calcium ion demonstrates potential for cross-linking with the protein,^25,26^ inducing the opposite effect to that intended with PEG. While plasticizers act to space the chains and increase the free volume, cross-linking agents perform the opposite function. Samples containing 0.05 g of calcium stearate have a concentration of 12.5 mmol/L, sufficient to form aggregates and interfere with plasticization.^27^ Both compounds that alter the WPIT formulation are amphipathic and can interact with both the polar and nonpolar parts of the protein, potentially leading to competition for binding sites.

Thus, another piece of evidence of the plastification process would be a decrease in the glass transition temperature (*T*_g_) of the samples compared to pure WPI, as it is expected that when plastification occurs, chain mobility increases.^6^ Despite that, it was not possible to identify the glass transition in the samples. Figure 6 displays the results obtained by DSC analysis.

**Figure 6:**
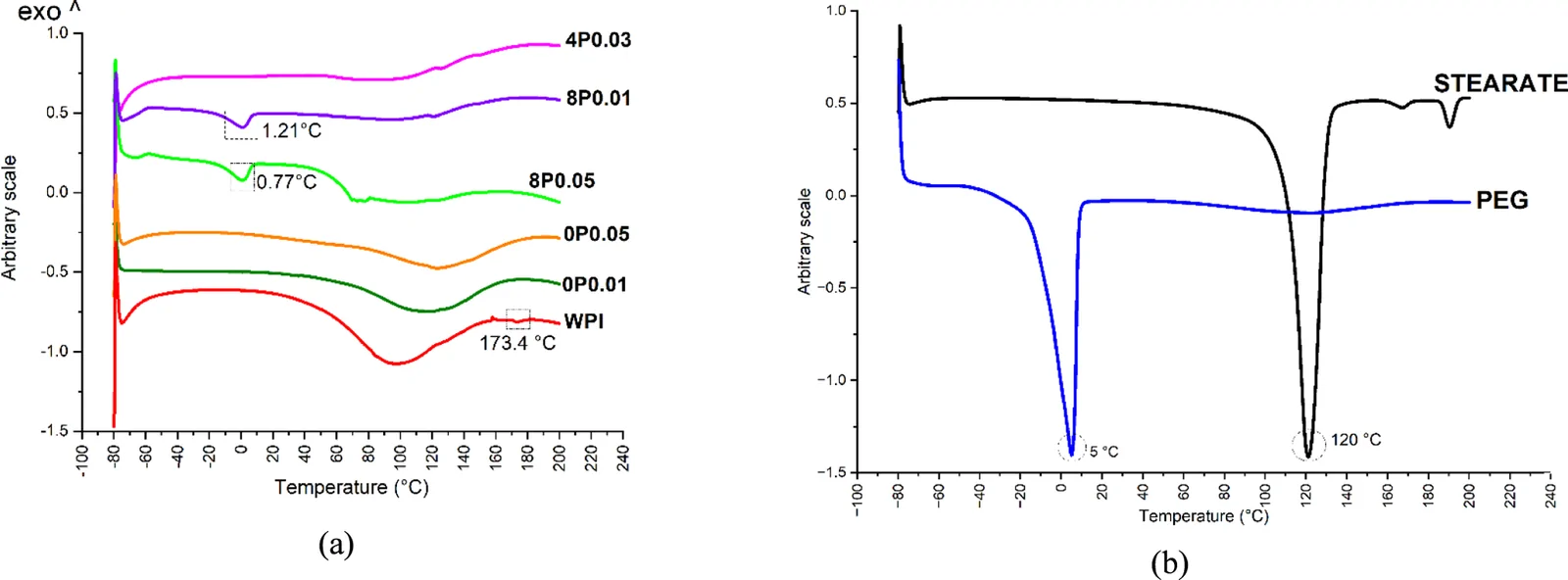
Results of DSC analyses: (a) samples and WPI; (b) PEG and calcium stearate.

In Figure 6a, a glass transition was identified in pure WPI at 173 °C, consistent with the typical range for native proteins (120–250 °C).^28^ However, this transition was not observed in any samples modified with PEG and calcium stearate. In samples 8P0.05 and 8P0.01, an endothermic event was observed at 0.77 and 1.21 °C, respectively, probably related to the melting of PEG, also visible in Figure 6b at 5 °C. The absence of this peak in sample 4P0.03 can be attributed to the lower PEG content. In Figure 6b, an endothermic peak at 120 °C indicates structural changes in the crystalline phase of calcium stearate into a smectic phase.^24^ In addition, endothermic peaks between 80 and 120 °C in the samples were associated with water loss,^29^ a process that occurs gradually as the depth and mobility of water molecules in the protein matrix increase.^30^

The moisture contents of the samples after drying, shown in Table 4, ranged from 8% to 12%. As seen in Figure 7a, calcium stearate presented a stronger influence on moisture content than PEG. This could be explained by the hydrophobic properties of the nonpolar part of the calcium stearate chain, which may repel water molecules. The interaction between PEG and calcium stearate is significant for moisture content, as shown in Figure 7b.

**Table 4:** Moisture Content by Sample

sample	moisture content (%)
**4P0.03**	8.2
**0P0.01**	12.5
**8P0.05**	11.1
**8P0.01**	10.9
**0P0.05**	9.3

**Figure 7:**
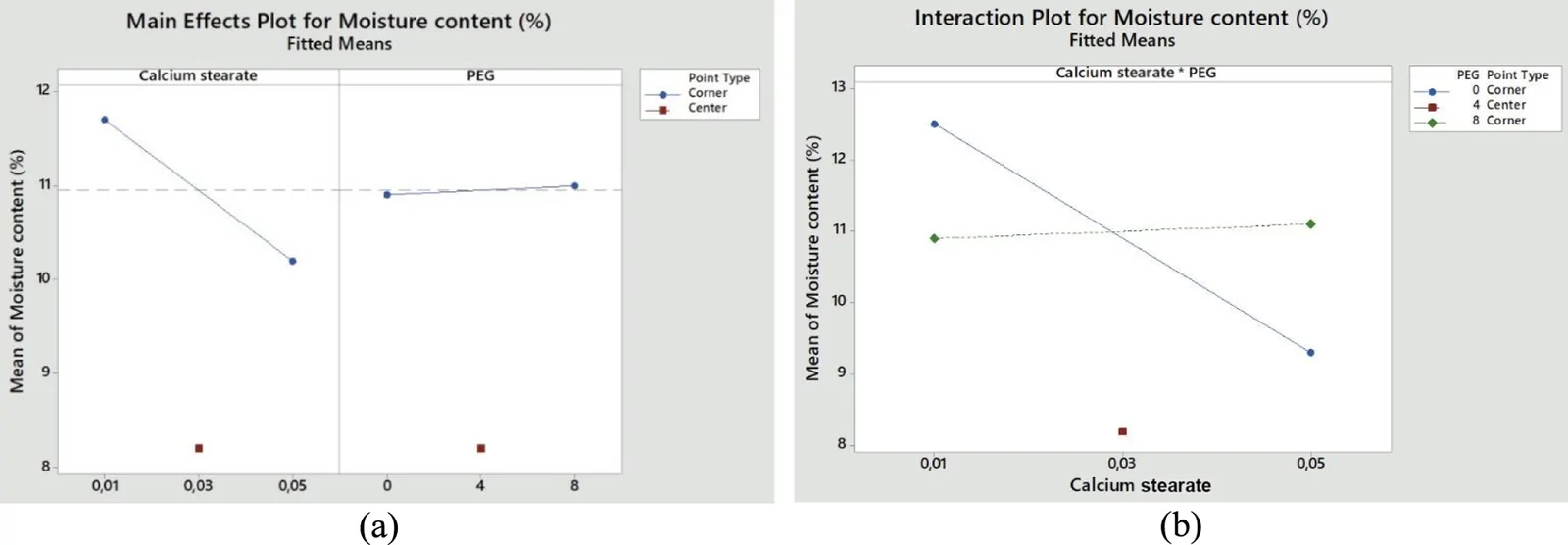
(A) Main effects plot of calcium stearate and PEG for moisture content; (b) Interaction plot of calcium stearate and PEG for moisture content.

Figure 8 shows the thermal behavior of the raw materials used in the formulation of the samples. Table 5 presents the onset temperature (*T*_onset_) and peak temperature (*T*_peak_), which correspond to the extrapolated temperature at the onset of the greatest mass loss event and the corresponding temperature of the greatest mass loss variation, respectively.^31^ For the thermal degradation of WPI, two stages are observed: the first, before 200 °C, refers to water loss, and the second, with greater mass loss at 256 °C, is related to the degradation of protein structures.^6,28^ *T*_onset_ is important for defining the safe processing window between *T*_g_ and the onset temperature of degradation.^28^ Similarly, calcium stearate exhibits two main degradation stages: water loss at around 100 °C and the onset of decomposition at 391 °C, associated with the degradation of carboxyl groups.^32^ PEG, on the other hand, has a single decomposition stage, starting at 237 °C, with a peak at 303 °C, due to the breaking of the −C–C and −C–O- bonds in its main chain.^33^

**Figure 8:**
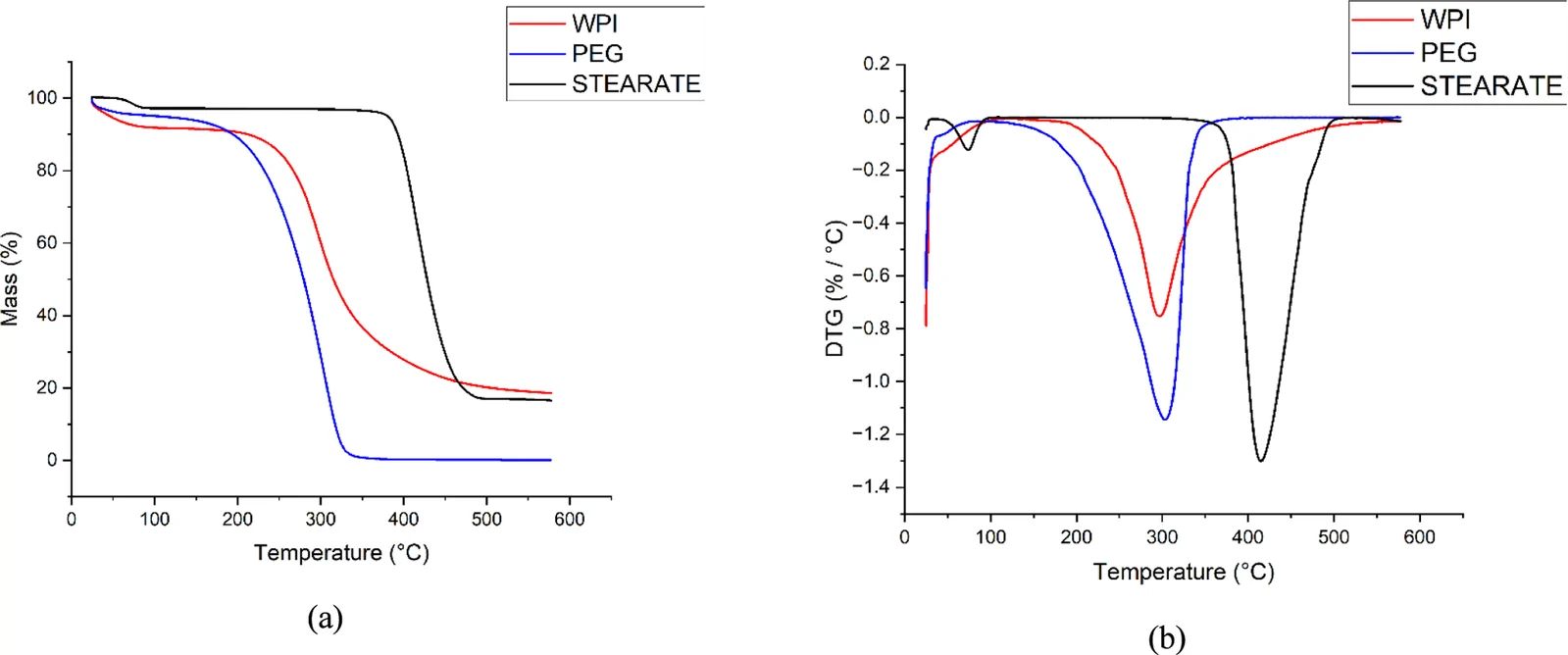
TGA results for raw materials. (a) Mass loss (%) by temperature. (b) Derivative Thermogravimetry (DTG) (%/°C) by temperature.

**Table 5:** TGA Results for Raw Materials and Samples

material/sample	*T*_onset_ (degC)	*T*_peak_ (degC)
WPI	256	296
PEG	237	303
calcium stearate	391	414
4P0.03	235	289
0P0.01	253	287
8P0.05	223	286
8P0.01	227	296
0P0.05	250	280

Figure 9 and Table 5 show the results of the thermal analysis of the samples by TGA. Samples with PEG presented a decrease in *T*_onset_. WPIT formulations also have the presence of sodium sulfite, which inhibits the formation of disulfide bonds and may have contributed to this lower thermal stability.^29^ The addition of plasticizers such as PEG can reduce the *T*_onset_, as it increases the mobility of polymer chains and decreases intermolecular interactions,^6^ requiring less energy for degradation, an effect already observed by Ramos et al.^14^ Thus, a decrease in *T*_onset_ was expected in formulations with PEG.^7^ PEG presents a stronger influence in *T*_onset_ than calcium stearate, as shown in Figure 10a, which is other evidence of its own plasticizing effect. The interaction of PEG and calcium stearate was not significant for *T*_onset_, as presented in Figure 10b.

**Figure 9:**
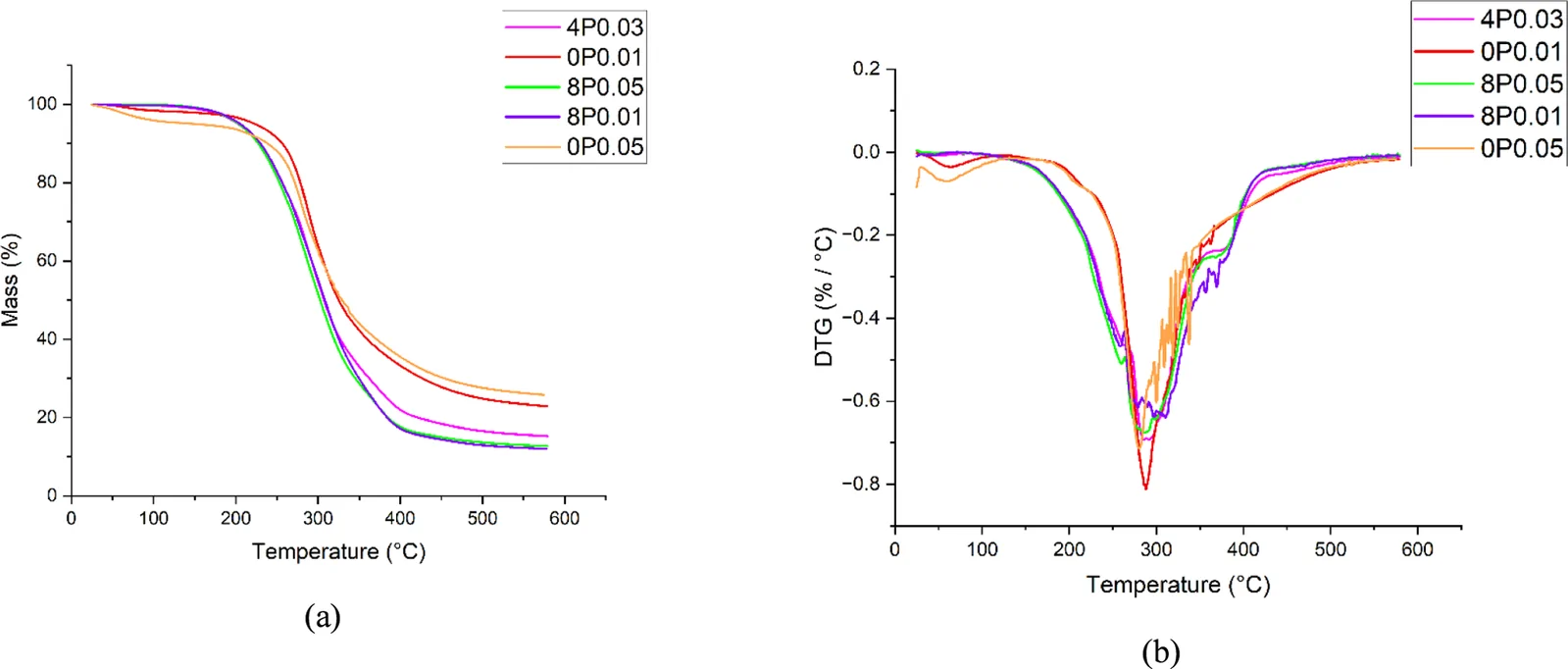
TGA results for samples: (a) Mass loss (%) by temperature; (b) Derivative Thermogravimetry (DTG) (%/°C) by temperature.

**Figure 10:**
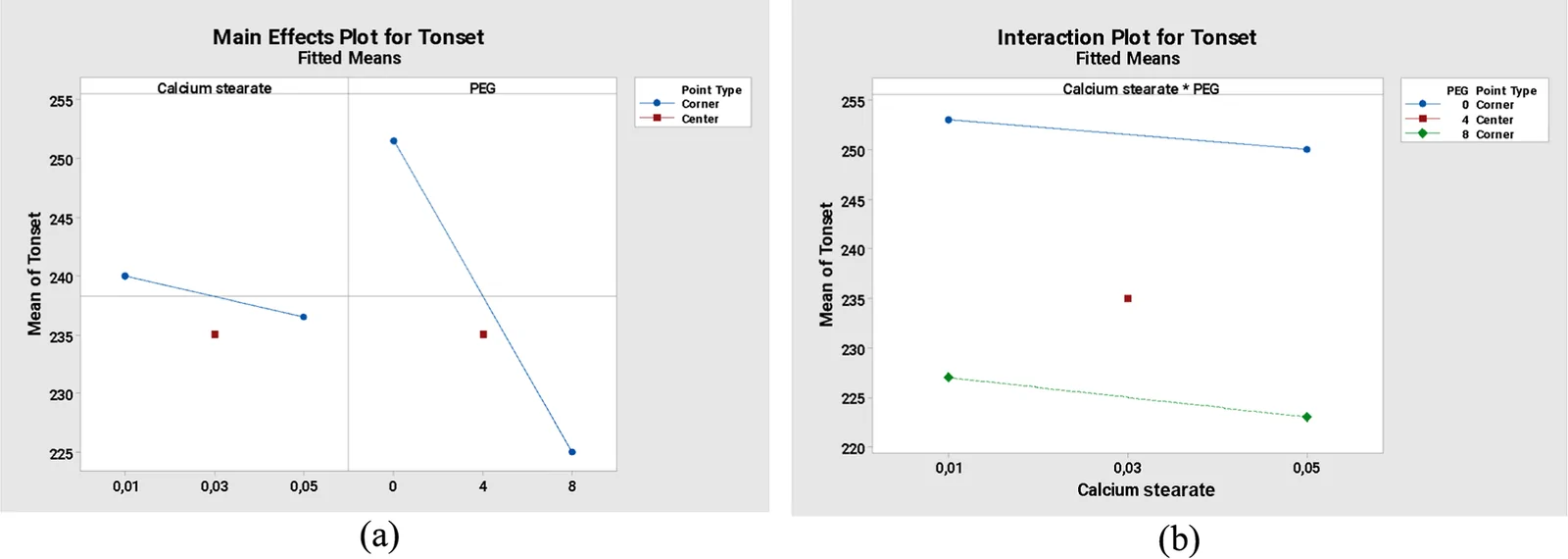
(A) Main effects plot of calcium stearate and PEG for *T*_onset_; (b) Interaction plot of calcium stearate and PEG for *T*_onset_.

The absence of complete plasticization of the samples, even in the presence of PEG, may be related to the presence of calcium ions from stearate.^25,26^ The smaller dimension of the Ca^2+^ ion favors its diffusion in aqueous media, allowing it to have greater interaction with protein chains. This can lead to the ion-bridging effect, which is responsible for the formation of gels in proteins. This effect occurs because calcium, being a bivalent ion, can bind to negatively charged sites in different protein chains. This cross-linking reduces the electrostatic repulsion between the chains and allows them to come closer together, contributing to the formation of three-dimensional networks.^26,34^ The formation of these structures, although useful in food and pharmaceutical applications,^25^ hinders processing by techniques such as extrusion.

WPI can form gels upon aqueous denaturation, with cohesion resulting from interactions between denatured protein chains, mainly intermolecular forces and disulfide bonds.^35^ Mulvhill and Kinsella^27^ indicate that the optimal concentration of Ca^2+^ for gel formation in β-lactoglobulin is 10 mM. Lower concentrations, around 5 mM, allow gel formation, but with less strength, while higher concentrations, close to 15 mM, result in excessive cross-linking, forming large, dense, and discontinuous aggregates. The calcium concentrations used in the formulations (2.5 to 12.5 mM) are within that range, as seen in Figure 11. Therefore, the presence of Ca^2+^ may have led to partial cross-linking of the proteins. Thus, some regions may have been plasticized, mainly with the presence of PEG, while others were cross-linked, explaining the duality observed in the analysis results.

**Figure 11:**
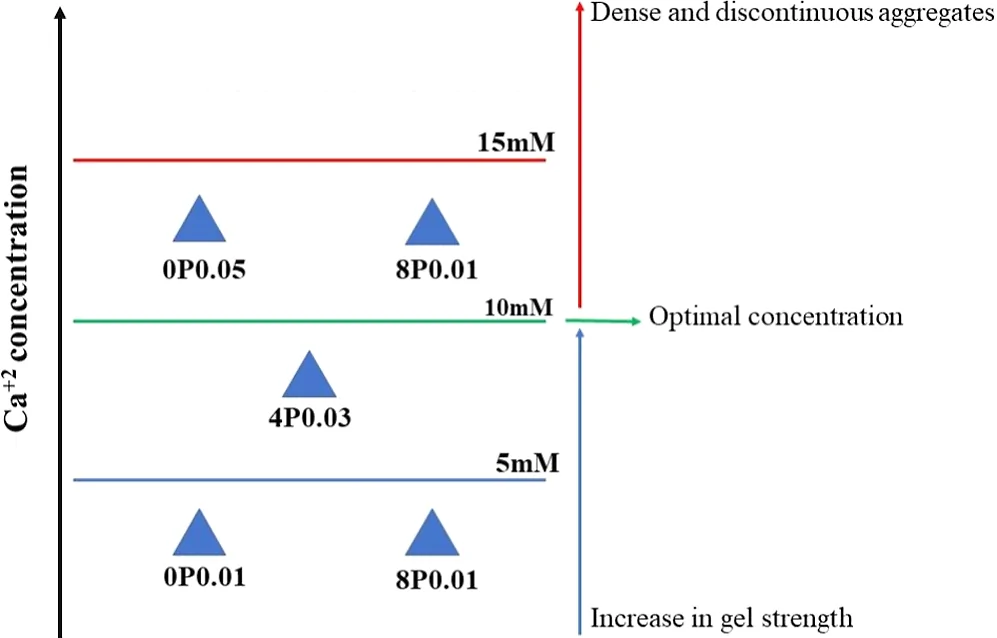
Concentrations of low, optimal, and excessive gel strength in WPIT samples. The triangles indicate the concentrations present in the samples studied.

## 4 Conclusions

The modified protein did not present complete plasticization, as the variations in α helix were small in comparison to pure WPI. Complex viscosity showed power law behavior and no Newtonian plateau in the terminal zone for WPIT samples, and evidence of cross-linking for the sample with the maximum calcium stearate and without PEG. Samples with calcium stearate and without PEG, and the center point presented crossover of storage and loss moduli in the terminal zone. GAP and complex viscosity were more influenced by calcium stearate than by PEG. It was not possible to obtain a *T*_g_ in the modified samples, as would be expected in plasticized systems. On the other hand, TGA analysis suggests a possible plasticizing action of PEG, which influenced the *T*_onset_ more than calcium stearate. Increasing PEG content led to a reduction in thermal stability, which could be related to an enhancement in chain mobility.

The presence of calcium stearate could form gels in the WPI structure during denaturation, as some WPIT formulations presented concentrations of this additive above 10 mM, which is optimal to form cross-links.
